# Mechanistic Insights into the Role of Molecular Chaperones in Protein Misfolding Diseases: From Molecular Recognition to Amyloid Disassembly

**DOI:** 10.3390/ijms21239186

**Published:** 2020-12-02

**Authors:** Rubén Hervás, Javier Oroz

**Affiliations:** 1 Stowers Institute for Medical Research, Kansas City, MO 64110, USA; ruh@stowers.org; 2 Rocasolano Institute for Physical Chemistry, Spanish National Research Council (IQFR-CSIC), Serrano 119, E-28006 Madrid, Spain

**Keywords:** amyloid aggregation, liquid–liquid phase separation, molecular chaperone, Hsp70, Hsp90, functional amyloid, toxic client, pro-toxic co-chaperone, aberrant condensate

## Abstract

Age-dependent alterations in the proteostasis network are crucial in the progress of prevalent neurodegenerative diseases, such as Alzheimer’s, Parkinson’s, or amyotrophic lateral sclerosis, which are characterized by the presence of insoluble protein deposits in degenerating neurons. Because molecular chaperones deter misfolded protein aggregation, regulate functional phase separation, and even dissolve noxious aggregates, they are considered major sentinels impeding the molecular processes that lead to cell damage in the course of these diseases. Indeed, members of the chaperome, such as molecular chaperones and co-chaperones, are increasingly recognized as therapeutic targets for the development of treatments against degenerative proteinopathies. Chaperones must recognize diverse toxic clients of different orders (soluble proteins, biomolecular condensates, organized protein aggregates). It is therefore critical to understand the basis of the selective chaperone recognition to discern the mechanisms of action of chaperones in protein conformational diseases. This review aimed to define the selective interplay between chaperones and toxic client proteins and the basis for the protective role of these interactions. The presence and availability of chaperone recognition motifs in soluble proteins and in insoluble aggregates, both functional and pathogenic, are discussed. Finally, the formation of aberrant (pro-toxic) chaperone complexes will also be disclosed.

## 1. Introduction

Optimal tissue and cellular maintenance require the preservation of protein homeostasis (proteostasis) [1]. These fine safeguarding processes are operated by an elaborate multiprotein and multicompartmental system called the proteostasis network (PN) [2]. The PN is in charge of securing proper protein synthesis, folding, maturation, and degradation. The activity of the PN, which is suited to the cellular requirements at different stages [1], is crucial for protecting cells against the extensive protein misfolding that arises under physiological and stress conditions, and therefore, alterations in the PN play pivotal roles in protein-misfolding diseases [2,3,4]. Because the PN’s activity is compromised in aging, age-related diseases are characterized by intracellular deposits of accumulated misfolded proteins [2,3]. Formation of these disease-related aggregates, usually of an amyloid nature, is triggered by aberrant structural transitions in monomeric functional proteins, which can display an ordered fold or remain intrinsically disordered [5]. Factors that facilitate aberrant protein structural conversions include genetic mutations and protein modifications, either in the aggregating proteins or in members of the PN [2], which, in combination with stress-induced and age-related PN decay, lead to protein aggregation processes that provoke cellular toxicity. 

Protein aggregation into amyloids [6] has been studied for decades, and many of the key factors governing the formation, composition, and structure of amyloids are established [5]. Remarkably, amyloids are not only related to the development of a large number of diseases but several functional amyloids have also been described in different organisms [7,8,9]. Disease-related amyloids have been considered hallmarks for many of the most devastating degenerative human disorders [5]. However, the concomitance between the presence of amyloid aggregates and disease progression is not always clear [10,11], and the nature of the toxic species in the aggregation cascades remains under debate [5,12,13,14]. Recent breakthroughs in the role of membraneless organelles formed by protein liquid–liquid phase separation (LLPS) in disease progression have brought dynamic condensates into the forefront [14,15,16]. Despite the abundant physiological roles of protein condensates [17], misfolded proteins [16] and pathogenic mutations [14] trigger the assembly into aberrant condensates related to toxicity, which are prevented by chaperone action [16]. In view of the critical role of molecular chaperones preventing aberrant LLPS and/or amyloid aggregation in diseases, understanding the fundamentals of chaperone recognition mechanisms is of utmost relevance for a mechanistic conception of protein misfolding diseases.

**Figure 1:**
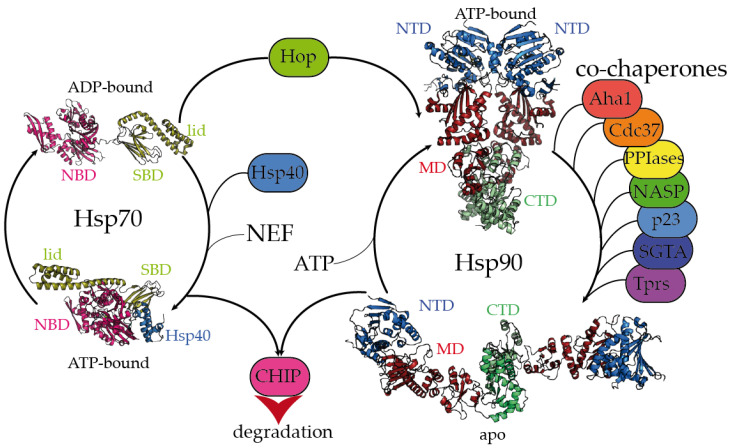
Hsp70 and Hsp90 chaperones are tightly regulated by co-chaperones. Hsp70 undergoes large allosteric changes upon ATP hydrolysis and substrate binding, from an extended, ADP-bound conformation with the substrate-binding domain (SBD) closed by the lid (PDB code 2kho) [45] to a collapsed, ATP-bound conformation with the SBD opened, favoring substrate release (PDB code 5nro) [46]. NBD stands for the nucleotide-binding domain. Hsp40 regulates the substrate folding and ATP hydrolysis by Hsp70 (Hsp40 J domain is included in PDB 5nro in the blue ribbon representation). NEF stands for the nucleotide exchange factor. Hop (Hsp-organizing protein) promotes the transfer of the substrate from the Hsp70 machinery to Hsp90. Hsp90 coexists in several conformations, from an extended, apo conformation [26] to a closed, nucleotide bound conformation, where the nucleotide-binding domains (NTD) would rotate to promote ATP hydrolysis [22]. CTD and MD stand for the C-terminal and middle domains, respectively. Hsp90′s activation cycle is closely regulated by co-chaperones [22]. Several representative human co-chaperones are included in the figure and are reviewed elsewhere [29,30]. Tprs stands for TPR-containing proteins. Phosphorylation of Hsp70 and Hsp90 C-terminal tails dictate the binding of CHIP, promoting substrate degradation [47].

Molecular chaperones play key roles in protein folding, aggregation, and degradation within the PN [2,3]. Because the chaperones’ function is tightly regulated and interconnected by a multitude of co-chaperones and other factors, this widespread protein network is collectively referred to as the “chaperome” [4,18]. The main molecular chaperones are characterized by a specific activation upon the heat shock response [19], which grants the proteins their names. Using this terminology, the main molecular chaperone nodes of the eukaryotic PN can be grouped into the DNAJ/Hsp40, Hsp70, Hsp90, Hsp100, chaperonin/Hsp60, and small Hsp (sHsp) [20,21,22]. Hsp70 and Hsp90 chaperones, which are evolutionary conserved and highly abundant in the cytosol [23], are well studied (Figure 1). Both chaperones undergo extensive conformational rearrangements upon ATP binding and hydrolysis [21,22,24]. While Hsp70 employs a restricted region of its structure for the transient recognition of short, mainly hydrophobic stretches of client proteins [24], client binding involves a large region of the Hsp90 structure [25,26]. Hsp70 and Hsp90 mainly work in a sequential arrangement, although they can bind clients simultaneously with the assistance of the co-chaperone Hop (Hsp-organizing protein) [23]. Client recognition specificity is modulated by a large array of co-chaperones (Figure 1). Hsp70 is mainly regulated by the versatile family of Hsp40/DNAJ chaperones [24,27,28] and assisted by sHsps [20], whereas Hsp90 associates with a large cohort of co-chaperones [22,29,30]. In short, Hsp70, Hsp90, and their co-chaperones are crucial members of the PN that are able to recognize misfolded proteins, aberrant condensates and protein aggregates, triaging proteins for refolding or degradation [2]. This review will cover crucial aspects of the mechanisms for client recognition employed by Hsp70 and Hsp90 chaperones that dictate their role in misfolding diseases, focusing on the principles that rule their interaction with monomeric misfolding intermediates, protein condensates, and amyloid aggregates.

## 2. Dynamic Hsp70 and Hsp90 Are Closely Monitored by Co-Chaperones 

The Hsp70 family of molecular chaperones is ubiquitously expressed and particularly relevant in the early stages of nascent polypeptide folding [2,3]. Human Hsp70 presents a variety of highly identical isoforms that coexist in cellular compartments, yet show functional specificity [31]. Hsp70 is composed of two domains, a 40 kDa, actin-like N-terminal nucleotide-binding domain (NBD) that binds ATP and regulates allostery and a 25 kDa C-terminal substrate-binding domain (SBD) (Figure 1). The substrate-binding pocket present in the SBD is capped by a lid, which undergoes large conformational changes following the ATP hydrolysis cycle toward the opening of the binding pocket and the release of the substrate (Figure 1). While the inherent ATPase activity of human Hsp70 is significantly slow, it is stimulated by the Hsp40/DNAJ family upon interaction with the J-domain, which additionally aids Hsp70 in the selective recruitment of clients [24,27,31]. Hsp40/DNAJ proteins interact differently with Hsp70 chaperones [28]. In the major class B, DNAJB1 exhibits a class-dependent autoinhibitory mechanism, in which a Gly/Phe-rich segment blocks the Hsp70-binding sites located in the J-domain. The presence of an additional Hsp70-binding site, which is not present in the class A Hsp40s, releases the Gly-Phe inhibition upon binding to the C-terminal IEEVD tail of Hsp70 [28]. In Hsp70, the NBD and SBD domains are separated by a highly conserved, dynamic, and amphipathic linker, which is crucial for the allosteric communication between both domains [24,32,33]. The sequence conservation between isoforms dramatically decays in the C-terminal region of the lid subdomain that closes the SBD, and in the following short C-terminal disordered fragment [32]. Interestingly, these variable regions determine the differential effects of Hsp70 isoforms on the aggregation or degradation of the Alzheimer’s-disease-related protein tau [34]. The very last C-terminal sequence of Hsp70 (containing the sequence IEEVD) is specifically recognized by the tetratricopeptide repeats (TPRs) present in the co-chaperone Hop, which escorts Hsp70:client complexes toward Hsp90 [35].

The biologically active Hsp90 dimer exhibits a tightly regulated equilibrium of extended and closed conformations (Figure 1) [22,26]. The ATPase activity is located in the N-terminal domain (NTD), which is highly conserved among the different Hsp90 isoforms [36]. The NTD is followed by a highly flexible, negatively charged linker, which is particularly relevant for client recognition [26,37,38]. The middle domain (MD) is important for allostery and co-chaperone and client binding, and the C-terminal domain (CTD) is critical for the dimerization and stabilization of Hsp90:client complexes [26]. Similar to Hsp70, Hsp90 has a conserved MEEVD motif at its C-terminus, which is targeted by TPR-containing proteins [26,35]. Human Hsp90 participates in the proper folding, maturation, and maintenance of a vast number of clients [23,39], and its activity, selectivity, and functionality are regulated by co-chaperones. Interestingly, the close inspection of all the Hsp90:co-chaperone complex structures described so far has revealed that co-chaperones that intervene at different stages of the Hsp90 activation cycle [22] bind to different interfaces of Hsp90, which delimits the available sites for the interaction with clients [26,37,40,41,42,43,44].

## 3. Selective Recognition of Misfolded Proteins by Hsp70 and Hsp90

Because Hsp70 recognizes short, hydrophobic sequences, and the availability and exposure of such regions trigger protein aggregation [3,5], the misregulation of Hsp70 and its co-chaperones can be related to the progress of protein misfolding diseases [48,49,50,51]. Indeed, pharmacological upregulation or overexpression of Hsp70 preserves viability in cells expressing aggregation-prone proteins, either by blocking the formation of toxic species [52,53,54,55] or by inducing aggregation into innocuous deposits [56]. Such sequences targeted by Hsp70 can be exposed as the result of partial misfolding of stably folded protein domains into metastable intermediates or present in protein sequences that remain intrinsically disordered. For instance, the protein transthyretin (TTR) forms a functional tetramer but can aggregate into insoluble deposits in TTR amyloidosis [57], causing neuropathy and cardiomyopathy [58]. During tetramer dissociation and misfolding, the C-terminal β-strand in the monomer of TTR unfolds, revealing hydrophobic segments [25] in a process that can be reverted by the protective T119M mutation [59]. However, it is still unknown whether Hsp70 directly binds misfolded TTR or whether the interaction would be mediated via an Hsp40 co-chaperone [31]. Although TTR deposits are mainly extracellular, they induce the activation of intracellular Hsp70, possibly through stimulation of the inflammatory response [60]. In a recent study, Masser and co-workers elegantly showed how Hsp70 specifically binds and inhibits Hsf1 (the transcription factor that induces the heat-shock response upon folding stress to balance proteostasis) [61]. Interestingly, increasing amounts of misfolded peptides in the cytosol during stress would titrate out Hsp70, releasing and activating Hsf1, which could ultimately lead to hyperstressed conditions and proteostasis failure [61].

The recognition of misfolded protein conformations by Hsp70 is not always thorough and may depend on the microenvironment. Hsp70 was found to bind specific conformations of human PrP prion protein in membrane microdomains in *Drosophila*, preventing the accumulation of misfolded conformers and alleviating PrP neurotoxicity [62]. Mutations in the superoxide dismutase 1 (SOD1) gene are related to familial cases of amyotrophic lateral sclerosis (ALS). Several mutations were found to reduce ion binding and promote partial misfolding in the cytosol [63]. One of these mutations, A4V, promotes the exposure of aggregation-prone regions, but not of Hsp70 recognition motifs (containing the sequences ^64^HFNPLSR^70^ and ^114^IGRTLVV^120^), providing an explanation for the accumulation of toxic SOD1 aggregates that result from incomplete recognition by Hsp70 [64,65]. The tumor suppressor p53 is another pertinent example of a misfolded target of Hsp70. Almost half of human cancers are associated with the loss of function and/or gain-of-toxic function of p53, which is triggered by its amyloid-like aggregation [66]. The Hsp70 recognition motif (^251^ILTII^255^ in the sequence of p53) is buried in the core of p53′s tridimensional structure [67,68]. The role of Hsp70 in regulating p53′s activity has remained controversial since some evidence indicates that Hsp70 induces p53 misfolding and cytoplasmic aggregation [69,70]. Interestingly, Hsp70 was found to trap misfolded intermediates of p53 with the assistance of Hsp40 co-chaperones, hence stabilizing aggregation-prone conformations [68]. The recruitment of Hsp90 and its co-chaperone Hop impedes the Hsp70-driven aggregation of p53 since Hop would promote the transfer of misfolded p53 to Hsp90. Hsp90 and Hop then restore the native state of p53 [68,71]. Therefore, proper recognition of misfolded motifs in pathogenic proteins by Hsp70 and its intricate coalition with Hsp90 and co-chaperones are key to preventing further aggregation and maintaining a balanced proteostasis. 

Hsp70 recognition motifs remain more exposed in intrinsically disordered proteins (IDPs). The natively disordered protein tau plays multiple roles in neurons but can form aggregates of different compositions in tauopathies [72]. Despite being highly soluble, tau contains several hydrophobic fragments (containing the sequences ^275^VQIINK^280^ and ^306^VQIVYK^311^), which mediate its amyloid aggregation [72] and are specifically recognized by Hsp70 [34]. Intriguingly, only the inducible variant of Hsp70 is able to promote proteasomal tau clearance, which is mediated by the co-chaperone ubiquitin ligase CHIP [34]. Polyglutamine expansions in the protein huntingtin (HTT) form cytoplasmic inclusion bodies (huntingtin bodies) and are causative for hereditary forms of Huntington’s disease [73]. Hsp70 specifically recognizes the sequence ahead of the polyglutamine tract in HTT (containing the sequence ^1^MATLEKLMKAFESLKSF^17^), which mediates intermolecular associations that are relevant for aggregation [74] and may be important for stabilizing helical conformations within the polyglutamine tract [75]. Mediated by this interaction, Hsp70 and Hsp40 inhibit HTT aggregation and alleviate the derived toxicity [74]. Human islet amyloid polypeptide (IAPP) is the major component of the amyloid deposits found in patients with non-insulin-dependent (type II) diabetes mellitus. Bongiovanni and coworkers showed that a modified version of Hsp70 specifically targeted the sequence ^56^FGAILSS^62^ in IAPP [76]. Remarkably, Hsp70 seemed to first accelerate IAPP aggregation before suppressing it, leading to reduced cytotoxicity [76]. The TAR DNA binding protein of 43 kDa (TDP-43) is abundantly found forming insoluble aggregates in familial and sporadic cases of ALS, frontotemporal lobar degeneration, and even Alzheimer’s disease and the recently defined Limbic-predominant age-related TDP-43 encephalopathy (LATE) [77,78]. Overexpression of Hsp70 reduced TDP-43 aggregation without promoting its degradation [79]. Hsp70 and Hsp40 co-chaperones were found to stably interact with the C-terminal disordered region of TDP-43, preserving TDP-43′s solubility and functionality [80]. Similar to the Hsf1 inhibition mechanism mediated by Hsp70 mentioned earlier [61], activation of stress would enhance protein misfolding in the cytosol, which would titrate out Hsp70 and Hsp40 from TDP-43, favoring its aggregation and loss of function [80]. Overall, Hsp70 in combination with DNAJ/Hsp40 co-chaperones are very potent neutralizers of protein aggregation, particularly for IDPs, through a wide variety of mechanisms. 

Evidence indicates that the interaction between Hsp90 and folded substrates are not restricted to a particular region of Hsp90 but cover a wide interface in the chaperone, leading to the formation of much more dynamic, pleomorphic, and multivalent complexes than in the case of Hsp70. For instance, in the absence of co-chaperones, Hsp90 forms multiple dynamic complexes with misfolded TTR and p53 [25,81,82]. Despite displaying selective recognition for misfolded TTR, Hsp90 was unable to promote the refolding of TTR in the absence of co-chaperones [25]. Conversely, the binding of Hsp90 promoted the interconversion between metastable conformers within an ensemble of conformations in p53 [81]. These dynamic modes of interactions are in apparent conflict with the structure of Hsp90 in a complex with the co-chaperone Cdc37 and the client Cdk4, which was solved using cryo-electron microscopy (cryo-EM) [37]. In that structure, Hsp90 traps a misfolded form of the client Cdk4 in a complex stabilized by the co-chaperone Cdc37. Therefore, questions are raised whether the presence of co-chaperones bound to Hsp90 would promote preferential modes of interaction for the client proteins. Nevertheless, evidence obtained from nuclear magnetic resonance (NMR) spectroscopy indicates that, in solution, co-chaperones bind to human Hsp90 in a very dynamic fashion, where fully bound forms of the Hsp90:co-chaperone complex coexist with a partially unbound co-chaperone [26,43].

**Figure 2:**
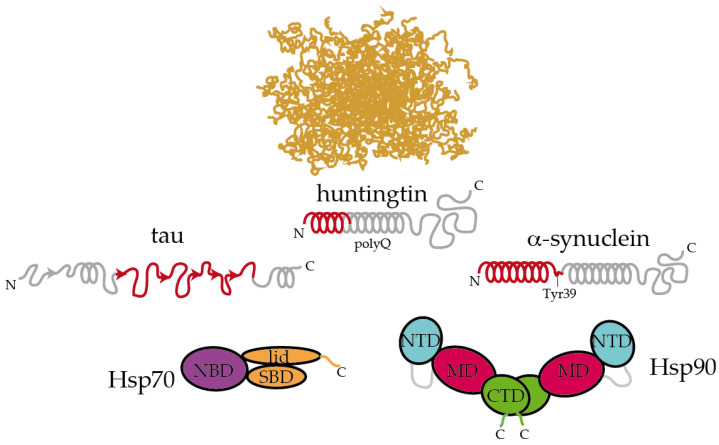
Hsp70 and Hsp90 recognize the structured moieties present in intrinsically disordered proteins (IDPs). A representative ensemble of conformations adopted by an IDP in solution is pictured at the top [94]. Tau, huntingtin, and α-synuclein contain elements that have a tendency to adopt a partial secondary structure (represented with helices and arrows for α-helices and β-strands, respectively) [83,88,89]. The moieties recognized by Hsp70 and Hsp90 are colored in red [26,34,86,89]. On tau, Hsp70 specifically recognizes the stretches ^275^VQIINK^280^, ^306^VQIVYK^311^, and ^375^KLTFRE^380^ [34]. The disordered C-terminal tails of Hsp70 and Hsp90 are represented. In Hsp90, the flexible, negatively charged linker connecting the NTD and the MD is represented with a grey line. The length of huntingtin is shortened for simplicity.

The interaction between Hsp90 and IDP clients is highly dynamic and polymorphic. In the case of the longest isoform of human tau (called htau40 [83]), a large fraction of the protein binds to Hsp90, irrespective of the allosteric state of the chaperone [26]. Specifically, the proline-rich region and microtubule-binding domains of tau are strongly involved in the interaction with Hsp90. Interestingly, these regions are rich in positively charged residues, which may well be trapped by the negatively charged linker between the NTD and MD domains of Hsp90 [38]. In addition, the aggregation-prone, hydrophobic stretches present in tau are specifically recognized by nonpolar patches exposed along the surface of the different domains of Hsp90, supporting the multivalent nature of the interaction [26]. These hydrophobic stretches in tau, which were also specifically recognized by Hsp70 [34], adopt a partial β-structure in solution [83], which might be relevant for the recognition by chaperones (Figure 2). Hence, multiple conformations of tau adhere along one arm of the Hsp90 dimer in a strikingly polymorphic, yet specific manner [26]. Quite compellingly, the presence of a co-chaperone bound to Hsp90 drastically reshapes the tau conformational ensemble in the complex [26], thus leading to the presumption that studying the structural consequences of chaperone:co-chaperone complexes on bound clients may be more biologically relevant. 

Inspecting the interaction between chaperones and IDPs reveals interesting insights. Extracellular senile plaques composed of amyloid-β (Aβ) peptide are also characteristic of Alzheimer’s disease [5]. Upon cleavage of the transmembrane amyloid precursor protein, amphipathic fragments of 40 and 42 residues (called Aβ40 and Aβ42) are released. Both Aβ40 and Aβ42 show structural transitions toward enrichment in a β-sheet structure and the propensity to aggregate [84]. Hsp90 and Hsp70:Hsp40 complexes could both interact with Aβ42 in the monomeric and oligomeric forms, triggering structural changes in Aβ42 that halt its further aggregation [85]. In a recent systematic study, Burmann and coworkers showed that several divergent chaperones, including Hsp70 and Hsp90, all recognized the same regions of monomeric α-synuclein [86]. α-Synuclein is the main constituent of the Lewy bodies, which are intracellular inclusions found in Parkinson’s disease [87]. More specifically, Hsp70 and Hsp90 recognized the N-terminal region containing the sequence ^1^MDVFMKGLSKAKEGVVAAAEKTKQGVAEAAGKTKE^35^, which has a tendency to adopt α-helical conformations [88], and the Tyr39 residue of α-synuclein. Interaction with Hsp70 and Hsp90 prevented α-synuclein’s oligomerization [86]. Interestingly, these interactions were found to be very transient in cells, and subtle changes in the cellular levels of α-synuclein or chaperones, modifications in α-synuclein or induction of cellular stress could dissociate the complexes and imbalance proteostasis, leading to α-synuclein aggregation and eventually causing Parkinson’s disease [86]. In a similar fashion, Hsp90 specifically binds to the identical region of HTT that is recognized by Hsp70 [74,89]. This particular region, which was proposed to form an amphiphatic α-helix in solution is able to bind to multiple regions of Hsp90 [89].

Altogether, it appears that Hsp70 and Hsp90 recognize the same repertoire of hydrophobic stretches present in client proteins, with Hsp90 showing an additional affinity for bulky aromatic residues. Remarkably, when these chaperone recognition motifs are present in IDPs, they show a significant tendency to adopt a secondary structure (Figure 2). This raises the question of whether chaperone recognition is based on sequential or structural motifs. It is possible that Hsp70 and Hsp90 selectively bind to one side of the α-helix or β-strand where most of the hydrophobic sidechains of the motif reside [89]. Besides being rich in hydrophobic residues, the regions that are recognized by Hsp70 and Hsp90 on IDPs are also significantly abundant in positively charged residues [26]. Therefore, the interaction between chaperones and IDPs is multivalent [26]. The outcome of these apparently similar recognition mechanisms by Hsp70 and Hsp90 on the triaging of the client protein can be drastically different, but this resolution seems to be dictated by the specific co-chaperone recruited to the complex (Figure 1). For instance, the co-chaperones Hop and CHIP bind to the same regions of Hsp70 and Hsp90, and the selection is determined by phosphorylation of the binding site in the chaperones [47]. While the binding of Hop enhances client protein folding, the binding of CHIP promotes client degradation [47]. Thus, because Hsp90 is shown to be ambivalent in client triaging, it has been considered to play a passive scaffolder role in the context of protein misfolding diseases. Indeed, several co-chaperones have been raised as promising therapeutic targets for various neurodegenerative diseases [26,90,91,92,93].

## 4. Hsp70 and Hsp90 Play Crucial Roles in Disease-Related LLPS

LLPS is a long-studied physicochemical process whose enormous impact in protein biochemistry and cell biology has been recognized recently [17,95]. During the last decades, major cellular membraneless compartments covering a wide range of functions have been discovered [95,96,97,98]. These highly dynamic species are formed by the biomolecular coacervation of usually proteins and RNA to typically keep them dormant during stressful conditions [17], and the molecules within the condensate can be exchanged from the condensed to the dispersed phases. Proteinaceous LLPS is governed by weak, transient, and reversible interactions [99], and a pattern of “stickers” (aromatic residues) and “spacers” (polar moieties) on proteins has been established to determine the phase behavior [100,101]. Because of their elevated dynamics and plasticity and patterning of stickers and spacers, IDPs are actively involved in LLPS [101]. Intriguingly, even though biomolecular condensates are involved in a plethora of physiological functions, there is a delicate equilibrium between the physiological and aberrant condensates in diseases [15], and condensates that are formed by several proteins were shown to evolve to fibrillar solid aggregates of an amyloid nature [102,103,104,105,106].

Biomolecular condensates are metastable states that quickly react to changes in the environment. Thus, tight regulation is required to modulate condensates’ disassembly or their progress to fibrillar structures (either functional or pathogenic) [15,107,108]. In aging, when the stressful conditions lead to an overwhelmed PN [2], IDPs within condensates, such as RNP (ribonucleoprotein particle) granules or stress granules (SGs), engage misfolded proteins that result from defective ribosomal products [109]. In contrast, the promiscuous recruitment of misfolded proteins in the granules is avoided by the PN in normal conditions [16,109]. Surprisingly, while physiological SGs are devoid of misfolded proteins, and therefore, not recognized by the PN or autophagy machineries, aberrant SGs are rich in misfolded proteins, attracting members of the PN and autophagy machineries [16,110,111]. Along these lines, age-dependent decay in the activity of the PN [2] promotes the accumulation of aberrant SGs, as has indeed been observed in organisms of increasing age [112].

Rather than being cleared by autophagy, it appears that condensates are preferably disassembled by the PN, favoring the cellular recycling of the components [110]. Condensates, such as SGs, accumulate many different elements of the PN [110]. For instance, Hsp70 was found to be essential for dissolving SGs to reactivate translation in *Drosophila* after a heat shock [108]. In contrast, Hsp90 was found to be a key player for the formation and maintenance of P-bodies, which are cytoplasmic granules where inactive mRNAs and translation repressors are stored in a dormant state [113]. The inhibition of Hsp90 induces the disassembly of P-bodies, reactivating translation [113]. In addition, the inhibition of Hsp90 promotes the disassembly of TDP-43-containing SGs and pathogenic aggregates [114]. This apparent contradictory effect of Hsp70 versus Hsp90 in condensate maintenance and disassembly must be interpreted with caution since the pharmacological inhibition of Hsp90 usually induces the concomitant overactivation of Hsp70 and other sHsps [114,115,116]. The chaperone complex formed by Hsp70, the co-chaperone BAG3, and the sHsp Hspb8 is crucial for dissolving SGs and prevent the accumulation of aberrant granules in ALS [111,117]. In a sort of stepwise mechanism, Hsp70 promotes the rapid disassembly of SGs, where persisting aberrant SGs and remaining aggregates are transported through microtubules to protein inclusions for autophagic degradation [16].

It remains unclear how and which species are recognized by the Hsp70/Hsp90 machineries within the condensates. The lingering of misfolded proteins in high concentrations that are segregated either in the core or the periphery of the condensate [16,118] could promote structural conversions toward the acquisition of β-structure and cross-seeding processes as a starting point for amyloid formation [15], where these motifs could become targets for chaperone recognition. Audas and coworkers recently described the A-body, a nuclear condensate that arrests amyloidogenic proteins to languish in their aggregation in response to stressors [119]. Upon termination of the stress conditions, Hsp70 and Hsp90 chaperones disassemble the A-bodies and avoid the further aggregation of the constituents inside these condensates [119]. In addition, because aberrant SGs also enclose misfolded ribosomal products, improper regulation of SGs by the PN would not only promote the aggregation of amyloid proteins but also impair the synthesis of many proteins that are fundamental for the adaptation to aging due to the lack of function of these arrested ribosomal products [110]. Therefore, the misregulation of physiological condensates by the PN in aging can entail dramatic consequences, and deciphering the exact mechanisms of chaperone-mediated condensate dissolution or maintenance is imperative in this emerging field of research.

## 5. Pathological vs. Functional Amyloids

**Figure 3:**
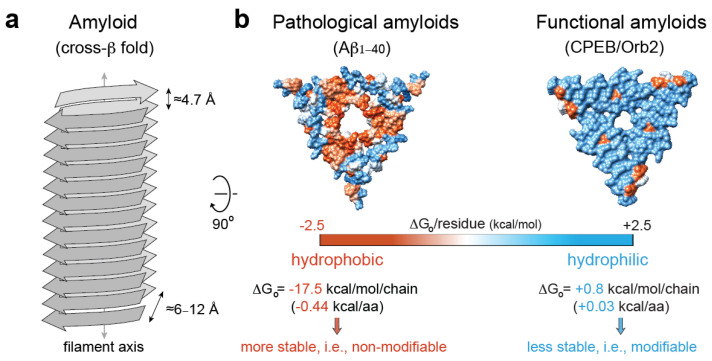
Pathological vs. functional amyloids. (**a**) Structural traits of the cross-β amyloid fold [123]. (**b**) Hydrophobicity map on the molecular surface of a representative pathological amyloid, namely, β-amyloid_1–40_ in Alzheimer’s disease brain tissue (PDB code 2m4j [152]), vs. a functional, neuronal amyloid implicated in memory persistence (PDB code 6vps [9]), showing the different stabilization energy. The hydrophobic residues are colored red, and the hydrophilic ones are colored blue. The solvation energy calculations were obtained from ref. [153].

Amyloids are unbranched, elongated filamentous protein aggregates that are characterized by a “cross-β” X-ray diffraction pattern, which arises from the cross-β sheet quaternary structure of the amyloid fold [120,121]. In the amyloid fold, which is considered to lie in the lowest energy level of the folding energy landscapes as extremely stable folds [122], β-stranded protein conformers are stacked along the filament axis via hydrogen bonds into a parallel or anti-parallel β-sheet. Usually, more than one β-sheet align in parallel and 6–12 Å apart, which produces a dominant diffraction signal on the equator of the X-ray diffraction pattern. Along the β-sheets, β-strands are arranged perpendicular to the major filament axis and are interspaced by ≈4.8 Å, which gives a strong, sharp diffraction signal on the meridional axis of the diffraction pattern [123] (Figure 3a). In addition to these specific structural features, amyloids are usually (i) resistant to proteases [124]; (ii) resistant to treatment with ionic detergents, such as sodium dodecyl sulfate [125]; (iii) upon binding to the Congo red dye, they exhibit an apple-to-green birefringence when viewed between crossed polarizers [126], while binding to the benzathiole dye Thioflavin-T produces a dramatic increase in fluorescence intensity [127].

Amyloid deposition has been associated with ≈50 human diseases. Particularly, in the human nervous system, amyloid deposition of widely expressed, multifunctional, and conformationally flexible proteins is a hallmark of cell-type-specific degenerative disorders in different regions of the brain [5]. However, the history of self-replicating amyloid states that operate in diverse biological phenomena, known as “functional amyloids,” is relatively short. Nonetheless, functional amyloids have been found to play key roles in several systems, including:

(i) Scaffolding systems, such as curli in microbial biofilm, providing a scaffold to protect bacteria and promote adherence to host cells [128].
(ii) Storage systems, such as Pmel17, which allows for the sequestration and condensation of melanin in the lumen of melanosomes [129]; vicilin, a 7S globulin in garden pea *Pisum sativum* L. seeds, which plays a crucial role in seed longevity [130]; peptide hormones for storage and release of certain hormones in the pituitary secretory granules [8]; Xvelo in *Xenopus* or Bucky Ball in zebrafish, which form a non-membranous compartment termed the Balbiani body to store germline-specific maternal mRNAs [131].
(iii) Signal transduction systems, such as the RIPK1-RIPK3 heterodimer, which controls necrotic signaling in mammals [132], or the fungal prion [HET-s], which regulates heterokaryon incompatibility between genetically similar fungi acting as a trigger for cell death activation [133].
(iv) Translation regulator systems, such as the yeast prion [PSI^+^], a general translational terminator that provides heritable phenotypic variability [134]; Rim4, a translation inhibitor of cyclin mRNA that controls gametogenesis [135]; or Orb2, the *Drosophila* member of the mRNA-binding cytoplasmic polyadenylation element family of proteins [136,137].
(v) Enzymatic systems, such as the membrane-associated protein Herzog (Hzg), which is required for the proper establishment of segment polarity in *Drosophila* embryo through an amyloid-like assembly that activates a phosphatase that is crucial for proper development [138].

Accordingly, although amyloids are widely associated with human disease, they are also utilized by diverse biological systems to control protein function in a context-dependent way. This versatility raises questions regarding the regulatory mechanisms used by the cell to disassemble pathological amyloids, and at the same time, to modulate the assembly and disassembly of functional amyloids when required. Understanding the structural differences between functional and pathological amyloids should provide valuable insights into these questions. Since the development of the landmark approach that is used to determine the atomic-resolution structures of amyloids [123], solid-state NMR and cryo-EM have revolutionized the high-resolution structure determination of patient-derived pathological amyloids in recent years [139,140,141,142,143]. By definition, across the amyloid formation pathways, several polymorphic amyloids with different maturity will be formed [5]. On top of that, an analysis of ex vivo samples revealed disease-specific amyloid structures for tau [139,140,141,142,143] and α-synuclein [144], as well as the presence of abundant hydrophobic interfaces that contribute to amyloid stability [145].

As opposed to the highly stable, hydrophobic amyloid cores of pathological amyloids, the core of functional amyloids is more versatile, with hydrophilic interfaces, kinked-β sheets [146], and charged residues that confer amyloid instability. For example, the translator regulator CPEB/Orb2, when isolated from adult fruit fly heads, forms a functional amyloid that is composed of a less stable, pH-dependent, hydrophilic amyloid core [9]. The prion-like protein [HET-s] from *Podospora anserine*, which assembles in vitro to form a left-handed β-solenoid composed of four in-register parallel β-sheets, is another representative example of a hydrophilic amyloid [147]. In addition, several human RNA-binding proteins implicated in the formation of dynamic SGs in response to cellular stress, such as hnRNPA1, hnRNPA2, or FUS, can form hydrophilic, reversible amyloids that are incompatible with the canonical dry-steric-zipper structure that is abundantly observed in pathological amyloids [148,149,150,151]. Altogether, these studies indicate that hydrophilic interfaces, which contribute with smaller stabilization solvation energy [151], could be associated with stable, yet hypothetically reversible, functional amyloids, while hydrophobic interfaces are abundant in irreversible, pathological amyloids [145] (Figure 3b).

## 6. Interplay between Chaperones and Amyloids

Given their high thermodynamic stability, it remains poorly understood as to what extent cellular regulatory mechanisms disassemble already existing pathological amyloids in vivo. As part of the PN, molecular chaperones not only interfere with early nucleation and amyloid elongation processes but also disassemble already existing pathological amyloids [5]. The Hsp70 disaggregation machinery (composed of Hsp70, Hsp40, and Hsp110-type nucleotide exchange factors; Figure 1) has the capacity to disassemble in vitro preformed amyloids of tau [154], α-synuclein [28,155,156,157], and HTT [55] with varying efficiencies. Furthermore, tau amyloid extracted from human Alzheimer’s brain tissue was disassembled by Hsp70, demonstrating that this disaggregation machinery is able to dissociate disease-relevant species [154]. Although the mechanistic basis of amyloid recognition and disassembly by the Hsp70 disaggregation machinery is still poorly understood, it promotes the release of monomers from in-vitro-assembled α-synuclein amyloids, possibly via two different modes of operation: amyloid fragmentation and end depolymerization [156]. Strikingly, amyloid disaggregation could have negative biological implications since small polymorphic fragments could recruit available monomers and seed novel amyloid formation [156]. Indeed, in vivo, the Hsp70 disaggregation activity increases the amyloid burden through the formation of toxic amyloid-type species of α-synuclein and polyglutamine expansions in *Caenorhabditis elegans* [158].

On its own, Hsp90 can disassemble in-vitro-preformed TDP-43 amyloids [159]. In collaboration with nicotinamide mononucleotide adenylyl transferases (NMNATs), Hsp90 can function as a neuroprotective chaperone, disaggregating and refolding previously aggregated proteins, such as tau, through an unclear mechanism in which Hsp90 might activate the C-terminal ATP-binding site of NMNAT, conferring to NMNAT a chaperone activity [160]. Consequently, further studies are necessary to clarify the exact role of the chaperone disaggregation machineries dissolving pathological amyloids and their role with toxicity in vivo, especially in the nervous system, since in postmitotic neurons, the amyloid load cannot be diluted by cell division.

On the other hand, a certain degree of reversibility is favorable for broader biological functions [3,5]. Thus, although functional amyloids share biophysical properties with pathological ones [5], in order to restrict functions both in space and time, the formation of a functional amyloid fold must arise in response to extra- or intracellular stimuli. These stimuli, although stable, must be dynamic or even reversible through regulatory mechanisms. For example, amyloid assembly makes Hzg a catalytically active phosphatase in a context-dependent way (i.e., during gastrulation), which implies the presence of still unknown regulatory processes for amyloid assembly, and perhaps disassembly, during embryonic patterning and tissue specification [138]. In this scenario, PN machineries could (i) allow or, when required, even promote functional amyloid assembly, such as the JJJ2, which is a DNAJ co-chaperone that has been found to enhance Orb2 aggregation and facilitate long-term memory formation, when exogenously expressed in *Drosophila* [161]; (ii) assist with amyloid disassembly once the function is performed, such as the Hsp104 chaperone, which has the ability to fragment [PSI^+^] amyloids into smaller fragments, and is required for prion propagation in yeast [162]. Remarkably, the hydrophilic nature of functional amyloids not only provides the necessary instability for functional turnover but might also have an impact on different regulatory mechanisms. For example, in Orb2, the protonation state of histidine residues due to pH changes could influence Orb2 amyloid stability, as has been suggested for other functional amyloids, such as mammalian Pmel17 [163,164] or bacterial curli [165]. Along these lines, the decrease in stability of functional amyloids compared to pathological amyloids can entail a faster dissociation rate, as this rate was determined to correlate with amyloid stability and, in general, with toxicity [166].

Mechanistically, it is well understood how Hsp70 binds extended substrates in a process that is tightly regulated by Hsp40 and NEF/Hsp110 (Figure 1). Still, how Hsp70 SBD can accommodate large folded states, such as amyloids, remains unclear. Structural evidence suggests that the *α*-helical lid subdomain can adopt largely different conformational states and does not entirely close over the bound substrate, presenting a more suitable scenario for amyloid recognition by Hsp70 [167]. The Hsp70 disaggregation machinery functional cycle occurs on amyloid surfaces and follows a nucleation model [157]. For *α*-synuclein disaggregation, DNAJB1 first recognizes aggregated *α*-synuclein through multivalent interactions with the flexible C-terminal tails (residues 123–129). Importantly, the motifs selected by Hsp40 that trigger its recruitment onto the amyloid surface remain largely unknown. The specific interaction of DNAJB1 with aggregated α-synuclein promotes the crowding of Hsp70 at the surface of the aggregate through multiple recruitment cycles. Here, Hsp70 interacts mostly with hydrophobic and lysine-rich regions located at the N terminus of *α*-synuclein (residues 1–10 and 37–43). Hsp110 potentiates *α*-synuclein amyloid disaggregation, acting together with DNAJB1 loading Hsp70 proteins in a densely packed arrangement at the aggregate surface, which is key for the generation of entropic-pulling forces that destabilize the amyloid fold [157,168,169]. In this process, the aforementioned DNAJB1 Gly-Phe auto-inhibition is key for efficient targeting and clustering of Hsp70 onto the surface of an *α*-synuclein amyloid [28]. While Hsp110 is key for aggregate disassembly, the Hsp40 family member availability drives the aggregate specificity [28,157,170]. Hence, it seems probable that structural variations in pathological and functional amyloids, such as the presence of hydrophobic versus hydrophilic interfaces (Figure 3), may be reflected in a diversity of different Hsp40 requirements, which provide flexible target selectivity, raising the possibility of the simultaneous disassembly of functional and pathological amyloids. Alternatively, the potential structural heterogeneity exhibited by the pathological and functional amyloid assemblies might require Hsp70/Hsp90-independent, alternative disaggregation activities, such as the recently reported RuvBL1/2, Cyp40, and HTRA1, which allow, through diverse mechanisms, amyloid disassembly in the metazoan cytoplasm [171,172,173].

## 7. Aberrant Chaperone Complexes Have Deleterious Consequences

Despite the abundant examples of chaperones halting aggregation or dissolving condensates, several chaperone complexes have been described as able to trigger pathogenic aggregation of the client proteins, therefore, displaying a pro-toxic role. The peptidyl-proline isomerase (PPIase) FKBP51 is a major Hsp90 co-chaperone that aids in the profolding of multiple substrates [22]. FKBP51 was found to be overexpressed in aged brains and, particularly, in Alzheimer’s brains, where it specifically synergized with Hsp90 to promote the pathogenic aggregation of tau [92]. When FKBP51 interacts in a symmetric manner with the Hsp90 dimer, the bound tau ensemble reshapes toward the nucleation of tau’s proline-rich region near FKBP51′s PPIase catalytic site [26], which would promote the *cis*–*trans* isomerization of the abundant proline residues in this particular region of tau. This reaction has a strong influence on the proper balance of tau hyperphosphorylation (and therefore, its pathogenic aggregation [72]) since phosphorylation of Thr residues promotes *cis* isomerization of the closest proline residue in the sequence due to spatial constraints [174]. Indeed, *cis*-proline, but not *trans*-proline, phosphorylated tau is found in protein deposits in the brains of humans with mild cognitive impairment [175]. PIN1 PPIase efficiently converts *cis*-proline into *trans*-proline and is considered a key safeguard PPIase against the pathogenic aggregation of tau [176]. Nonetheless, during Alzheimer’s disease progression, PIN1 function is downregulated, which is weighed by the upregulation of the highly similar PPIase FKBP51 [91,176]. This compensatory upregulation of FKBP51 has a dramatic downside since FKBP51 binds Hsp90 with a high affinity via its TPR repeats [26], which will compete with the binding of the CHIP co-chaperone to Hsp90. The removal of CHIP uncouples the proteasomal clearance of Hsp90 clients, leading ultimately to the aberrant aggregation of tau [92]. Still, because FKBP51 bears *cis*–*trans* PPIase activity, which would in principle modulate the hyperphosphorylation of tau [176], the puzzling pro-toxic role displayed by FKBP51 on tau is still not fully understood. In addition, following a recent hypothesis postulating that the catalytic PPIase activity would be only a minor role of PPIases in aiding the folding activity of Hsp90 [177], it could well be that the transfiguration that Hsp90:FKBP51 induces on tau eliminates the interactions established between different regions of tau that protect the protein from aggregation [83,178]. Several other Hsp90 co-chaperones, such as Aha1, were also found to be elevated in Alzheimer’s brains, and to promote tau deposition and neurotoxicity, while the levels of protective co-chaperones were reduced, or even repressed, in aging and Alzheimer’s brains [179]. Thus, besides the abovementioned age-related PN decay, the balancing of the expression levels of members of the chaperome regulate the toxic aggregation in the cell and may contribute to disease onset and progression.

In a similar scenario, an excess of client recognition may lead to collateral damage. Increasing evidence of co-localization between tau and TDP-43 in amyloid deposits found in dementia brains suggest comorbid tau and TDP-43 pathologies [180]. Despite evidence of an interaction between tau and TDP-43 that may facilitate pathogenic conversions in one or both proteins [180,181], excessive recruitment of the chaperome by one of the proteins could also cause an imbalance in the triaging of the other protein assisted by the PN. Hsp90 in complex with the co-chaperone Cdc37 is responsible for the preservation of inactive TDP-43 in the nucleus [90]. The removal of Cdc37 promotes the proteolytic cleavage of TDP-43 and its transfer via Hsp90 to the autophagic machinery for degradation. Strikingly, the accumulation of tau in the cytosol would hijack Hsp90, ultimately leading to the accumulation of cleaved TDP-43 [90]. Somehow similarly, Hsp90:HTT interaction recruits the deubiquitinating enzyme ubiquitin-specific protease 19 (USP19), which promotes the upregulation of HTT and subsequently enhances its aggregation [89]. Indeed, the inhibition of the Hsp90:HTT interaction attenuates the upregulation of HTT mediated by USP19. Polyglutamine-expanded HTT aggregates could, therefore, hijack Hsp90 and Hsp70, causing a collapse of the proteostasis [89], which would impair several elementary cellular functions [182,183].

In a somewhat simplified view, it has been proposed that a combined therapeutic strategy that includes the inhibition of Hsp90 and potentiation of Hsp70 would provide neuroprotection based on the assumption that Hsp90 stabilizes the clients and Hsp70 promotes their degradation [184]. We have provided several examples documenting the fact that Hsp90 and Hsp70 offer a wide palette of effects on protein misfolding diseases, which suggests that the targets for therapeutic intervention should probably be focused more succinctly on other members of the chaperome. The inhibition of Hsp90 has been shown to be effective in Alzheimer’s, Parkinson’s, and Huntington’s diseases, as well as stroke and autoimmune encephalomyelitis [185,186,187,188]. However, because Hsp90 is an elementary member of the PN involved in the regulation of multiple cellular functions, its inhibition causes the overactivation of the heat shock response during extended periods, which may give rise to deleterious effects in the cell or exacerbated pro-inflammatory responses [76,184,189], as well as acquired resistance to the inhibitor [190]. Along these lines, targeting specific chaperone:co-chaperone complexes appear to be more promising for developing treatments for devastating neurodegenerative diseases [26,34,90,91,92,93]. For instance, Hsp70 paralogs showing a higher affinity for the co-chaperone Hsph2 showed potent degradation of an ALS-related mutant SOD1, while those Hsp70 paralogs preferring the co-chaperone Hop promoted SOD1 toxic aggregation [191]. In addition, while Hsp70 is responsible for suppressing α-synuclein aggregation, a single point modification on its partner Hsp40 can mute Hsp70′s action, leading to the deleterious accumulation of α-synuclein [192]. Hence, despite decades of awareness of the potency of chaperones as valuable targets for protein misfolding diseases [189], a deeper understanding of the interplay of the large variety of co-chaperones is key for developing strategic therapeutic approaches. In addition to the large complexity of the chaperome, natural polymorphisms may exist in humans [193]. Therefore, further research is needed in order to adequately manipulate the PN in age-related diseases.

## 8. Concluding Remarks

Hsp70 and Hsp90 chaperones have become primary therapeutic targets for a range of age-related diseases, which entail huge socio-economic impacts. Nonetheless, the interplay of chaperones and protein misfolding, LLPS, or aggregation is intricate and strongly regulated by co-chaperones. In this review, we present compelling evidence showing that co-chaperones may play leading roles in regulating protein misfolding and should be systematically included in mechanistic studies toward the understanding of pathogenic protein aggregation. Thus, in the future, we expect more members of the chaperome to be considered as potential therapeutic targets for proteinopathies with increased selectivity, as well as including natural polymorphisms, which may dictate different susceptibilities toward the diseases between individuals. In addition, many protein misfolding diseases feature context specificity, especially in those affecting the nervous system, where specific cell types are affected in each disease. Deciphering how the cell utilizes a cohort of chaperones or co-chaperones in a context-dependent manner, such as aging, can help us understand how cell-specific protein conformational diseases may arise. High-resolution structural studies are imperative for the understanding of the basis of the chaperone mode of action in the context of protein misfolding diseases. Because the proteins undergoing pathogenic structural conversions, condensation, or aggregation, as well as the members of the chaperome, are highly dynamic and form large proteinaceous assemblies, an integrative structural biology approach is required for their detailed comprehension. We anticipate that the combination of state-of-the-art NMR spectroscopy that is employed on soluble complexes and in biomolecular condensates, along with cryo-EM on ordered aggregates, will provide breakthroughs in the molecular mechanisms of protein misfolding diseases during the next exciting decades.

## Abbreviations

PN	Proteostasis network
LLPS	Liquid–liquid phase separation
Hsp	Heat-shock protein
sHsp	Small Hsp
NBD	Nucleotide-binding domain (Hsp70)
SBD	Substrate-binding domain (Hsp70)
TPR	Tetratricopeptide repeat
Hop	Hsp-organizing protein
NTD	N-terminal domain (Hsp90)
MD	Middle domain (Hsp90)
CTD	C-terminal domain (Hsp90)
NEF	Nucleotide exchange factor
ATP	Adenosine triphosphate
TTR	Transthyretin
SOD1	Superoxide dismutase 1
ALS	Amyotrophic lateral sclerosis
IDP	Intrinsically disordered protein
HTT	Huntingtin
IAPP	Islet amyloid polypeptide
TDP-43	TAR DNA binding protein of 43 kDa
NMR	Nuclear magnetic resonance
Aβ	Amyloid-β
RNP	Ribonucleoprotein particle
SG	Stress granule
Hzg	Herzog
NMNAT	Nicotinamide mononucleotide adenylyl transferase
PPIase	Peptidyl-proline isomerase
USP19	Ubiquitin-specific protease 19
